# Pediatrics

**Published:** 1898-04

**Authors:** Maud Josephine Frye

**Affiliations:** Physician to the babies’ ward, Erie County Hospital


### PEDIATRICS.

CONDUCTED BY MAUD JOSEPHINE FRYE, M. D.,
Physician to the babies' ward, Erie County Hospital.

### INTUSSUSCEPTION FOLLOWING DYSENTERY.

HULETT, H. L., of Allentown, N. Y., (*Archives of Pediatrics*, December, 1897,) reports an interesting case in a child of fifteen months, resulting in recovery. The child was ill for about a week with a sharp attack of dysentery, accompanied by tenesmus, which could not be entirely controlled. Twelve days after the onset of the illness the baby was suddenly taken worse, with all the symptoms of intussusception—namely, vomiting, soon becoming fecal, small bloody passages, colic, and the appearance of a sausage-shaped tumor in the region of the descending colon. Surgical interference was not deemed advisable on account of the child's extreme prostration. The treatment which eventually saved the child's life was high hot water injections every six hours, tincture of opium to the limit, brandy with white of egg and, later, peptonised milk as nourishment. The child was kept in a reclining position on the right side, the hips being elevated.

---

### MILK POISONING DUE TO PASTEURISED MILK.

KOPLIK (*Medical Record*, February 19, 1898,) takes ground against the use of Pasteurised milk as a food for infants and children. Pasteurisation of milk is a process which did not obtain the sanction of the master whose name it bears. Germs will always exist in milk obtained even from model dairies. The milk given to the vast number of our infants in large cities will be milk which, even if free from bacteria of disease, like tuberculosis, diphtheria, typhoid fever and scarlet fever, will still have a supply of bacteria indigenous to the milk, which must always be reckoned with. These are the bacteria to be considered as etiological factors in the causation of gastro-intestinal disorders of infancy and childhood. The most deleterious of these germs are not affected by Pasteurisation as at present carried out.

There develop in certain cases fed on Pasteurised milk a distinct set of symptoms, sometimes insidious, sometimes marked and troublesome, to which Koplik applies the term "milk-poisoning." In many cases the first symptom will be a looseness of the bowels. Children who have had only one movement or a constipated movement will suddenly have five, six or seven loose movements. At first the movements will have a yellow, curdled, lumpy appearance and a distinct acid odor, or they will have an admixture of green and an intensely disagreeable odor. Such an infant is restless, has colicky pains and a slight rise or no rise of temperature. In other cases the baby, who is apparently in good health, with three or four normal stools a day, will have a large number of greenish, ill-smelling movements of a very acid character and then after a dose of oil will relapse to a normal state. In other cases children will have one to three normal movements a day, followed by a large semifluid movement of extremely acid character and a distinctly penetrating sour odor. After such a movement the patient feels weak. In some cases the movements are simply increased in number for that patient. They are normal in color and odor, but more fluid than they should be, and the patient does not seem to increase in weight. There may be a slight rise of temperature toward evening. The treatment for all these cases is the substitution of sterilised for Pasteurised milk.

So far as a study of waste products can teach, Dr. Koplik has found little difference in the digestibility of raw, Pasteurised or sterilised milk, continuously prepared or modified in the same way, when administered to the same infant.

Sterilisation in the household must be carried on in a closed steam steriliser, where the temperature reaches 100° C. or close to this point. The milk should be maintained at this temperature for at least ten minutes. The milk should be cooled and then kept on ice or in a refrigerator below 20° C. until given to the infant.

---

### DIFFICULT DEFECATION IN INFANTS.

MARTIN (*Jour. Am. Med. Ass'n*, February 19, 1898,) gives the result of some interesting post mortem work on the anatomy of the rectum and sigmoid in infancy. It is generally recognised that the infant strains at stool. Dr. Martin finds the reason in the imperfect development of the anatomic features concerned in the mechanism of expulsion. These are as follows: The infant's lower gut is muscularly deficient. Its mobility within the abdomen, due to relatively long peritoneal attachments, is obstructive to defecation. The rectal valves appear to bear the same proportion to the gut in both adult and infant, but owing to lack of muscular development they offer disproportionate resistance. The infant's anus not being sufficiently expansible is also obstructive. For the normally formed infant the physician will find the solution of the problem of difficult defecation in the solution of the stool.

---

### SOME NEW METHODS OF RESUSCITATING STILL-BORN AND FEEBLE-BORN INFANTS.

BEDFORD BROWN (*Therapeutic Gazette*, June 15, 1897,) presents some valuable suggestions on this subject. He defines the still-born as an infant presenting all the indications of suspended animation, but capable of resuscitation. The condition may be distinguished from the dead-born by the rapid fall of the rectal temperature to 10° or 15° below normal in the latter case. Until such a fall of temperature is observed in the apparently dead-born child, efforts of resuscitation should be continued. An additional sign is the pupil. In the dead-born it is widely dilated; in the still-born but little if at all relaxed. The method of treatment advocated by the writer is to inject into each arm by hypodermic syringe four or five drops of whisky and a single drop of tincture of belladonna. This is done whether the infant be apparently dead or only feeble-born. If following this there is only a feeble response, or none at all, a drachm or two of warm, sterilised water is injected under the skin and about two drachms with a drop of aromatic spirits of ammonia into the intestines. If there is still no reaction and the temperature continues to decline while these measures are in progress, it is a fair test of the absence of vitality.

In the case of the still-born, very soon after the hypodermic the eyes suddenly open, the respiratory muscles are brought into action and the shrill cry of the new-born follows. Then cardiac action develops, the pulse becoming perceptible at the wrist and the cyanosis disappearing. The infant is then wrapped up well in warm flannel or cotton wool and placed on a hot water bag. The hypodermics may be repeated if necessary. The author cites some interesting cases showing the practical value of his method.

